# Incidental Meckel’s Diverticulum With Neuroendocrine Tumor

**DOI:** 10.7759/cureus.27625

**Published:** 2022-08-02

**Authors:** Clarissa K Chan, Tiffany Pham, Yash V Bhagat, William Fulton, Majid Kianmajd

**Affiliations:** 1 Surgery, NorthBay Medical Center, Fairfield, USA; 2 Internal Medicine, NorthBay Medical Center, Fairfield, USA; 3 Internal Medicine, University of Maryland Midtown Campus, Baltimore, USA; 4 Human Genetics, University of Calfornia, Los Angeles, USA

**Keywords:** carcinoid tumour, gastrointestinal neuroendocrine tumor, gastrointestinal carcinoid tumor, meckel´s diverticulum, incidental meckel's diverticulum

## Abstract

Meckel’s diverticulum (MD), the most common congenital disease of the small bowel, commonly presents with symptoms of painless rectal bleeding and intestinal obstruction. The treatment of symptomatic MD involves resection of the lesion regardless of patient age; however, the excision of asymptomatic and incidentally identified MDs in adults remain controversial. On one hand, the complications arising from MDs decrease with age, leading to a lower benefit than risk ratio with prophylactic resection. On the other hand, malignancies, such as neuroendocrine tumors, may arise over time from untreated MDs. This can lead to poor prognostic complications, such as liver or lymph node metastases. In this case report, we describe an incidental Meckel’s diverticulum discovered during an exploratory laparotomy for acute sigmoid diverticulitis in an adult male. Later biopsy findings discovered the lesion to contain a grade 1 neuroendocrine tumor. Based on our literature review findings, resection of the incidental Meckel's diverticulum was a reasonable approach given the low complication risks of the procedure and the possibility of malignant transformation and progression.

## Introduction

Meckel’s diverticulum (MD) is the most common congenital disease of the small bowel, affecting approximately 2% of the general population [1]. It is a diverticulum generally found about 100 centimeters proximal to the ileocecal valve as a result of incomplete obliteration of the vitelline (omphalomesenteric) duct during the 5th to 8th week of gestation [1]. As a true diverticulum, it contains all three layers of the normal intestinal wall: the mucosa, muscular, and serosa. This true diverticulum can sometimes contain ectopic tissue, most commonly gastric mucosa (80-85%), but it is also a site for neuroendocrine tumors (NETs; previously known as carcinoid tumors).

Symptoms from MD generally occur within the first two years of life, with 25-50% of patients that present symptoms being under 10 years of age [2,3]. The most common symptom is painless bleeding per rectum from ectopic gastric mucosa secreting acid leading to ulceration of the small bowel. Other symptoms include intestinal obstruction via volvulus or intussusception and diverticulitis within the MD [2]. In the pediatric population, the current recommendation is to remove an MD regardless of symptoms due to the increased risk of complications [4]. In contrast, the majority of MD cases in adults are asymptomatic and are often incidentally found in radiographic imaging [4]. The estimated complication risk ranges from 2-4% with the incidence of overall complications decreasing with increasing age. Thus, in the adult population, evidence-based treatment for an incidental finding of MD in the adult population remains controversial.

In this case report, we describe a patient with acute sigmoid diverticulitis who underwent an exploratory laparotomy where an incidental Meckel’s diverticulum was prophylactically excised. Pathology reports discovered the specimen to contain a grade 1 neuroendocrine tumor.

## Case presentation

A 54-year-old male presented to an emergency department with constant, diffuse abdominal pain associated with vomiting for five days. The patient reported severe, sharp lower abdominal pain. He also had chills, nausea, vomiting, and diarrhea. After three days, the abdominal pain and diarrhea subsided, at which point he became constipated. His last bowel movement was two days prior to admission, and he was still able to pass flatus. He denied fever, hematemesis, hematochezia, and melena. The patient has a history of hypertension and hyperlipidemia but no personal or family history of neoplastic disease. He had no previous episodes of diverticular disease and no previous colonoscopies.

The patient was admitted for possible bowel obstruction from an abdominal X-ray that was ordered by his primary care provider (PCP) prior to arrival. He was placed NPO (nothing by mouth) with a nasogastric tube and started on intravenous piperacillin/tazobactam. Patient vitals showed elevated blood pressure, with initial labs indicating neutrophil leukocytosis, mild hyponatremia, and hypokalemia (Table 1). Clinical examination revealed moderate tenderness to the lower quadrants with deep palpation, mild distention, and hypoactive bowel sounds.

**Table 1:** Patient Vital Signs and Laboratory Values on Admission

Patient Vital Signs and Laboratory Values on Admission	
Blood pressure	161/100 mmHg
Heart rate	93 beats/min
Respiratory rate	16 breaths/min
Oxygen saturation	98% on room air
White blood cell count	11.73 thousand/mm³
Absolute neutrophil count	9.04 x 10⁹/L
Sodium	131 mEq/L
Potassium	3.1 mEq/L

**Figure 1:**
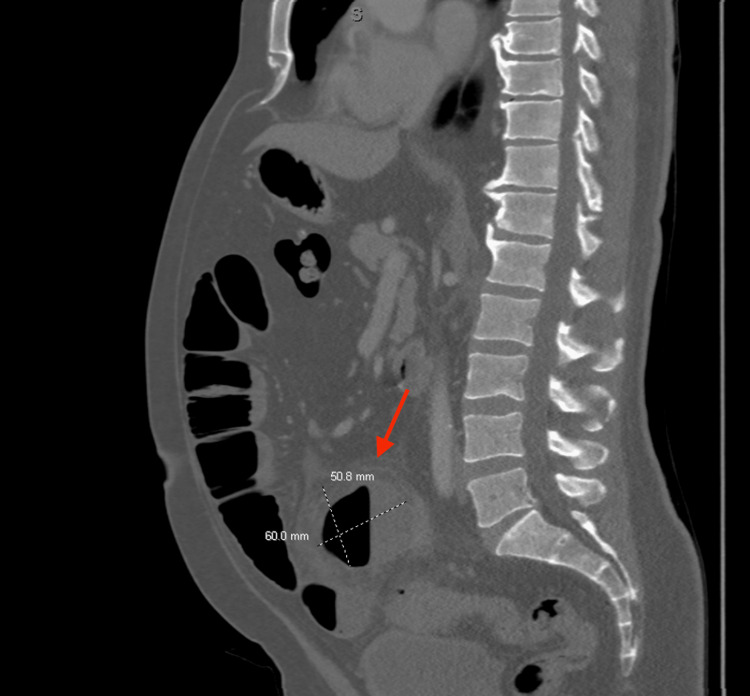
Sagittal View CT abdomen/pelvis with IV contrast CT: Computed Tomography; IV: Intravenous The CT image shows an abscess (50.8 x 60.0 x 60.0 mm, red arrow) with surrounding inflammation of the small bowel and sigmoid colon

**Figure 2:**
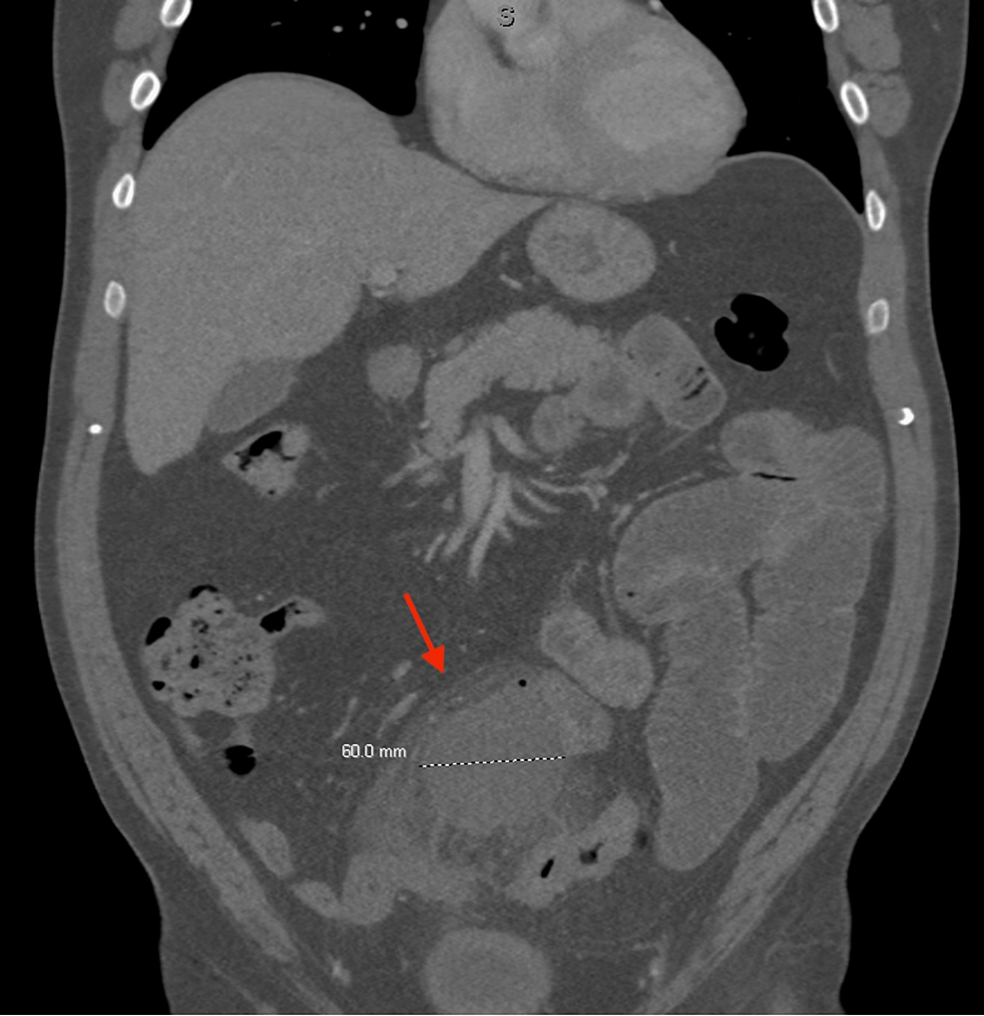
Coronal View of CT abdomen/pelvis with IV contrast CT: Computed tomography; IV: Intravenous The image depicts an abscess (50.8 x 60.0 x 60.0 mm, red arrow) with surrounding inflammation of the small bowel and sigmoid colon

A CT abdomen/pelvis with intravenous (IV) contrast was completed on hospital day one showing evidence of acute sigmoid diverticulitis, small bowel obstruction involving the jejunum and proximal ileum, a 50.8 x 60.0 x 60.0 mm abscess in the lower central abdomen with surrounding inflammation adjacent to the small bowel and sigmoid colon without pneumoperitoneum (Figures 1-2). 

On hospital day four, the patient remained hemodynamically stable with no fever or hypotension. However, his WBC increased to 15.92. His abdominal pain had improved, and he was still passing flatus. Repeat CT abdominal scan on hospital day three was unchanged. Due to the abscess being between loops of the bowel, interventional radiology decided it was unsafe to access. General surgery scheduled the patient for laparoscopy due to lack of abscess resolution despite antibiotics and inability to pass bowel movements.

The patient underwent robotic laparoscopy on hospital day five that was converted to an exploratory laparotomy due to extensive inflammation and the adhesions causing immobility of the small bowel and sigmoid colon. The 60 mm peritoneal abscess was located in the pelvis between two loops of the small bowel and was subsequently drained. In addition, the section of the terminal ileum containing the interloop abscess was severely inflamed and resected. For his sigmoid diverticulitis, low anterior sigmoidectomy with anastomosis was performed. Inspection of abdominal contents revealed an incidental Meckel’s diverticulum in the terminal ileum, which was excised with a GIA stapler and sent for pathology.

By postoperative day three/hospital day eight, the patient was able to pass flatus and bowel movements. The nasogastric tube clamping trial was adequate and was thus discontinued the next day with the patient tolerating a clear liquid diet. The patient was discharged postoperative day five/hospital day 10.

The colon resection that was sent to pathology revealed mucosa with diverticulitis and perforation in the background of diverticulosis with two benign lymph nodes. However, pathological examination of the small bowel Meckel’s diverticulum specimen contained a grade 1 neuroendocrine tumor Stage I (pT1, Nx, M0) that was, ​​3 mm in size, with resection margins negative for malignancy. The patient was therefore given an oncology referral.

After the surgery, the patient followed up with oncology. Based on the lack of symptoms (i.e. flushing, diarrhea, wheezing, bronchoconstriction) and negative resection margins, no further treatment was recommended. A multi-phase hepatic MRI was ordered to evaluate for liver lesions in the near future. Oncology also recommended to obtain a yearly CT chest/abdomen/pelvis and clinically monitor for any symptoms of hormone secretion, such as flushing, diarrhea, or bronchoconstriction. If symptoms develop, the next steps include obtaining a 24-hour plasma 5-HIAA (5-hydroxyindoleacetic acid) test.

## Discussion

To summarize, our case report described a 54-year-old male who underwent a robotic laparoscopy converted to exploratory laparotomy for acute sigmoid diverticulitis. An incidental MD was discovered and prophylactically resected, discovered by pathology to contain a grade 1 neuroendocrine tumor.

The definition and classification of carcinoid tumors, known as neuroendocrine tumors (NETs), have changed with increasing knowledge of their presenting features. Carcinoid tumors were originally used to refer to intestinal tumors that behaved less aggressively than common intestinal adenocarcinomas. Over the last century, it was discovered that they arose from enterochromaffin cells and secreted serotonin. Carcinoid tumors were later used to describe neuroendocrine tumors in the “lung, pancreas, and gut” [4]. In 1994, a group of pathologists proposed a revised classification, replacing the term carcinoid with “neuroendocrine tumor” to designate the entirety of neoplasms with neuroendocrine features [4]. NETs are currently defined as tumors containing cells that express general neuroendocrine marker proteins and cell-type-specific hormonal products. They are classified into three grades based on mitotic rate (low, intermediate, and high grade).

NETs are the most common tumors in the small intestine, most notably in the ileum or the appendix. They can also manifest within MDs, such as in our patient’s case. Common symptoms include intermittent abdominal pain, gastrointestinal bleeding, and obstruction. 10-20% of patients can also manifest carcinoid syndrome, with acute episodes of flushing, diarrhea, and asthma attacks. Although NETs are malignant tumors, they demonstrate low aggressiveness, with 70-80% of individuals being asymptomatic [5]. Due to the non-specific symptoms, particularly in the early stages of the disease, it takes between 2-20 years from the onset of symptoms to reach the final diagnosis. Half of the patients are in advanced stages of the disease with dissemination by the time they are finally diagnosed.

Despite MD being the most prevalent congenital disease of the small bowel, it is still an uncommon finding. Finding NETs within an MD is even rarer. Only 0.5%-3.2% of MD involve primary malignancies such as NETs, making it a relatively rare finding [1]. Though this is the case, the Surveillance, Epidemiology, and End Results (SEER) Program of the National Cancer Institute published in 1997 that there had been 163 reported total cases of MD malignancies, 121 of which were NETs [6]. NETs were therefore the most common malignancy arising from Meckel’s diverticulum (76.5% of cases), followed by adenocarcinoma (11.4%), gastrointestinal stromal tumor/leiomyosarcoma and sarcoma (10.8%), and lymphoma (1.3%). These findings indicate that MD is an important location of primary neuroendocrine tumors (NETs), which was noted in our patient.

Due to the low overall incidence of NET found within MD, many publications are often presented as case reports or retrospectives reviews with conflicting findings. As such, there seems to be no general consensus or evidence-based guidelines when faced with an incidental MD.

There is evidence supporting that the risk of excision may outweigh the benefits when considering the relatively high morbidity and mortality rates associated with removal. A 1976 review by Soltero and Bill reviewed 202 cases of complicated MDs that underwent emergency treatment. They calculated that the complications in MD carriers decreased from 4.2% at birth to 0% once adulthood was reached. Considering that there was a mortality rate of 6-7% for complicated MD surgery, this led them to conclude that in adulthood, 800 prophylactic resections would be necessary to prevent one death. As a result, they recommended that in the absence of specific risk factors, the high risk of post-op complications and low incidence of diverticulum-related complications in adults did not justify the removal of incidentally-discovered MD [7]. A more recent 2008 review from Zani et al. found similar results [3]. They noted a 5.3% incidence of complications based on 2,975 incidental MD patients that underwent prophylactic diverticulectomy. This is compared to a 1.3% incidence in those with conservatively treated MD. They, therefore, argued that 758 prophylactic diverticulectomies in incidentally detected MD patients would be needed to prevent one death in a patient with MD [7].

On the other hand, there is also evidence supporting lower morbidity and mortality rates in their retrospective reviews. Cullen et al. published a review in 1994 supporting prophylactic diverticulectomy. In comparison to the 0% in adults noted by Soltero and Bill, they calculated that the lifetime risk of complications without resection was 6.4%. The operative mortality and morbidity of prophylactic diverticulectomy for incidental, asymptomatic MD were only 1-2% and 2%, respectively. Once MD complications arose, however, the operative mortality and morbidity for diverticulectomy increased respectively to 2% and 12%. Therefore, they concluded that prophylactic surgical excision of MD was indicated at any age, especially before the age of 80 [6].

In 2005, Park et al. recommended a more conditional approach. They reviewed 1476 MD cases from 1950-2002 in Kings County, Washington with 16% of the patients symptomatic. While they did not recommend resection for all asymptomatic MD patients, they advised that resection should be performed in patients with specific associative factors that would increase future complication rates. Resection should be considered if patients were (1) male, (2) 50 years old or younger (3) diverticulum over 2 cm, and/or (4) had histologically abnormal tissue. Finding four, three, two, or one feature in a patient would be associated with complication rates of 70%, 42%, 25%, and 17%, respectively [2].

Extra consideration should also be taken for the possibility that NETs can be found in MD and cause serious complications for patients if intervention is delayed. In 2011, Thirunavukarasu et al. compared 163 cases of MD malignancies with 6214 cases of non-Meckelian ileal cancer, with 121 of the MD malignancies being NETs. They noted that MD is a high-risk area for cancer in the ileum, with the adjusted risk of cancer in MD at least 70 times higher than in all other ileal locations. Median overall survival for patients with any MD malignancy was 173 months with one-and five-year relative survival rates of 85.8% and 75.8%, respectively, depicting the slow-growing nature of the malignancy [8]. However, there is a high chance of metastases from NETs found within an MD. In a retrospective analysis of 280 NETs in MD from 2004-2015, researchers noted that 87 of the patients exhibited regional lymph node involvement. Thirty-nine of the 87 patients with lymph node involvement (44.8%) harbored node metastases, allowing them to conclude that regional lymph node involvement was associated with the presence of distant mets (p<0.001) [9]. Various recent chart reviews and case studies have also noted mesenteric lymph node metastases as well as liver metastases once NETs are discovered in MD, and patients have a poor prognosis when they are often diagnosed at an advanced stage [10,11]. In our patient’s case, regional lymphadenectomy was not performed since the MD was an incidental finding.

In regard to our case, the patient had two features that placed him at an increased risk for future complications, based on the findings of Park et al. [2]. The patient was (1) male and (2) over 50 years old. Overall, resection of the MD was most likely beneficial given that it contained an ectopic neuroendocrine tumor. His post-operative recovery within the hospital was uncomplicated and was discharged within five days of his surgery with full bowel function and normal appetite. The patient continues to be asymptomatic without any signs of metastatic disease.

As noted, there is a modest amount of published literature regarding treatment for NET in MD. One must, however, take into consideration that they are not randomized-control trials. This type of study design may not be feasible, even in the future, due to the relatively overall low incidence of NET in MD. Because of this, most are retrospective studies. These studies also include data and case reports that were published several decades ago in order to achieve a larger statistical power. Surgical sterility, the use of less invasive procedures, and technological advances have decreased the risk of post-surgical complications. This may play a role in decreasing mortality and morbidity risk of MD excision compared to surgeries performed over 50 years ago. Further research using more recent data in adult patients with incidental MD is required [12].

In addition, further research and discussion is needed regarding the method and extent of surgical intervention needed for patients with NETs in MD. Due to the downstream risk of metastases, there needs to be more consideration for the best method to resect MD (e.g., transverse diverticulectomy vs wedge resection vs segmental resection). There is also discussion on whether regional lymphadenectomy, liver debulking, or small bowel resection is necessary if there is a high chance of metastasis [10,11].

## Conclusions

In our case report, a 54-year-old male had an incidental, asymptomatic MD resected during an exploratory laparotomy for acute sigmoid diverticulitis, which was later found to contain a stage 1 neuroendocrine tumor. Currently, there is conflicting evidence regarding the risks and benefits of asymptomatic MD resection in adults. Based on our report and the current literature, resection of the incidental Meckel’s diverticulum was a reasonable approach due to the low complication risks of resection, the possibility of malignant transformation, and metastatic complications with increasing patient age.
